# A Novel Agnostic Respiratory Stimulant as a Treatment for Apnea of Prematurity: A Proof-of-Concept Study

**DOI:** 10.7759/cureus.28900

**Published:** 2022-09-07

**Authors:** Thomas L Miller, Lynn M Raab, Thomas H Shaffer, Alfred Schweikert, Frank Diana, Prem Fort, Alana S Frum, Joseph Pergolizzi, Robert B Raffa

**Affiliations:** 1 Pediatrics, Sidney Kimmel Medical College, Thomas Jefferson University, Philadelphia, USA; 2 Pediatrics, Enalare Therapeutics, Inc., Princeton, USA; 3 Veterinary Medicine, Thomas D. Morris, Inc., Reisterstown, USA; 4 Pediatrics, Lewis Katz School of Medicine, Temple University, Philadelphia, USA; 5 Pediatrics, Nemours Children's Health System, Wilmington, USA; 6 Respiratory Medicine, Enalare Therapeutics, Inc., Princeton, USA; 7 Neonatology, Johns Hopkins All Children's Hospital, St. Petersburg, USA; 8 Pediatrics, The Johns Hopkins University School of Medicine, Baltimore, USA; 9 Veterinary Medicine, The National Aquarium, Baltimore, USA; 10 Cardiology, Native Cardio Inc., Naples, USA; 11 Pharmacology, Temple University, Tucson, USA

**Keywords:** ventilatory control, carotid body, bk channel, premature lamb model, apnea of prematurity, ena-001

## Abstract

Aim/Objective: ENA-001 is a novel selective antagonist of large-conductance BK (big potassium) channels located in the carotid bodies, where they act as chemoreceptors that sense low arterial oxygen levels and establish a feedback loop to brainstem nuclei responsible for initiating spontaneous breathing and maintaining adequate oxygen to tissues. ENA-001 attenuates respiratory depression induced by a variety of chemical agents, essentially "agnostic" to the precipitating drug (e.g., opioid(s), benzodiazepine, alcohol, or propofol). But it had not been tested against respiratory depression resulting from a physiological cause, such as apnea of prematurity (AOP). This proof-of-principle study used a well-described animal model (premature lamb) to test the effectiveness of ENA-001 in the setting of an under-developed respiratory control system, similar to that in human AOP.

Materials and Methods: A set of twin lambs was delivered prematurely via caesarian section at 135 ± 2 d gestational age (GA). An arterial catheter was connected to a transducer for pressure monitoring and a venous catheter was connected to a pump for continuous infusion of 5% dextrose in water (D5W). Lambs were to receive four mechanical breaths for lung recruitment and then started on continuous positive airway pressure (CPAP). After a stabilization period of 15 minutes, the protocol called for the first lamb to be started on continuous infusion of ENA-001, with ascending dose hourly (0.4, 1.1, 2.0, 12.0 mg/kg/hr), while the second lamb was to serve as a sham (D5W) control. At least 10 representative breaths free of artifact from motion or atypical breaths were recorded using a pulmonary function system designed for neonatal research. To maintain a stable plane of anesthesia, repeat doses of fentanyl (1 µg IM) were given as needed based on blood pressure response to stimulation.

Results: Two male lambs were delivered. Unexpectedly, neither lamb exhibited a drive for spontaneous breathing. Each required manual ventilation, with a complete absence of spontaneous effort. Despite the poor prognosis owing to the absence of ventilatory effort, continuous infusion of the first dose of ENA-001 was started 20 minutes after birth. The test animal continued to require manual ventilation, which was continued for an additional 10 minutes. An intravenous (IV) bolus of ENA-001 was given. Nearly instantaneously following the delivery of the IV bolus, the lamb began breathing spontaneously and did not require manual intervention for the remainder of the study. The sham animal was delivered approximately an hour following the test animal. As with the test animal, the sham animal lacked spontaneous breathing efforts. A decision was made to manually ventilate for 30 minutes to match the course for the test animal. At the 30-minute time point, an IV bolus infusion of ENA-001 was delivered. Nearly instantaneously following the delivery of the IV bolus, the lamb began breathing spontaneously. After several minutes, the spontaneous breathing efforts abated, and manual ventilation was resumed. The animal was then sacrificed for tissue harvest.

Conclusion: These results suggest that ENA-001 might be an effective therapy, alone or as a co-medication, for the treatment of AOP. They further suggest that ENA-001 might have broader applications in situations of neurological ventilatory insufficiency.

## Introduction

Apnea of prematurity (AOP) is a respiratory disorder that afflicts many preterm infants, and it is one of the most common diagnoses in the neonatal intensive care unit (NICU). AOP results primarily from the physiologic immaturity of the respiratory system rather than a disease, a distinction important for the selection of a possible therapeutic intervention [1]. It is particularly attributable to the immature state of the respiratory control system [2]. The official definition of AOP varies by country and medical society, but has most recently been defined by the American Academy of Pediatrics Committee of Fetus and Newborn (AAP-COFN) as the occurrence of respiratory pauses of more than 20 seconds, or shorter breathing pauses with oxygen desaturation or cyanosis or bradycardia of less than 100 bpm in those of gestational age (GA) less than 37 weeks [3]. The incidence of AOP is inversely correlated to gestational age and birth weight; it occurs in more than half (54%) of those born at 30 to 31 weeks GA compared to 7% of those born at 34-35 weeks GA [4], and it is almost universal in preterm infants born at less than 29 weeks GA [5]. 

Current options for treating AOP are non-specific and have shortcomings. Although generally considered safe, there is still potential for adverse effects such as tachycardia and seizures [6]. Methylxanthines, such as aminophylline or more commonly caffeine are routinely administered for preterm neonates at risk for, or diagnosed with, AOP. Although generally effective in attenuating the frequency and severity of AOP [7], caffeine dosing is side-effect limited due to its central nervous system effects. Patients that continue to have events even after initiation of appropriate doses of caffeine often are given a trial of doxapram, an agent that acts on peripheral chemoreceptors, but that is not recommended for neonates, which highlights the need for alternative therapy. 

There are no approved therapies for AOP that target peripheral stimulation of the respiratory control loop. Such therapy could work independently of, or perhaps synergistically with, caffeine. ENA-001 is a new chemical entity being developed as a peripheral respiratory stimulant with a unique mechanism of action, targeting big potassium (BK) channels in the carotid bodies, mimicking the effect of hypoxia-induced afferent signaling feedback to the brain stem. ENA-001 is being studied for a number of indications related to respiratory insufficiencies, such as opioid-induced respiratory depression and post-operative respiratory distress. Clinical studies in adults, in addition to preclinical data in animals, have demonstrated that ENA-001 (formerly GAL-021 before acquisition by Enalare Therapeutics, Inc.) increases mammalian ventilatory drive in normoxia, as well as increases the sensitivity of the response to hypoxia [8,9]. These positive effects in both normoxia and hypoxia are likely to be relevant to AOP. 

The objective of the present study was to establish a proof-of-concept in the preterm lamb model of respiratory immaturity [10-12] that ENA-001 is able to impact respiratory drive in the immature neural circuitry in a manner that is translational to human AOP. Positive results in this study will form the basis to guide further protocol development using the preterm lamb model to determine an optimal dosing regimen as a function of GA, and the potential for substitute or adjunct treatment of AOP with caffeine as the standard of care. 

## Materials and methods

The protocol described one set of twin lambs to be delivered premature via cesarian section and stabilized as previously described [10-12], then managed for four hours. Based on experience with the model, the investigators targeted 135 ± 2 d GA, approximately 90% of the average gestational period for the lamb, as it was expected that the lambs would have a spontaneous respiratory drive and, therefore, even the control animal could be managed for four hours with continuous positive airway pressure (CPAP) and not require mechanical ventilation. The protocol was reviewed and approved by the Institutional Animal Care and Use Committee (IACUC) at Thomas D. Morris, Inc., the contract research organization where the study was conducted (approval number 22-004). 

A standard flank caesarian section was performed and the first lamb’s head was exteriorized and covered with a glove filled with warm saline to prevent aspiration of room air. Utilizing local anesthesia (1 ml; 2% lidocaine), a midline incision at the midpoint of the anterior neck was made to facilitate instrumentation with a 3.5mm endotracheal tube, as well as cannulating the common carotid artery and external jugular vein using 5Fr catheters. Once the umbilical cord was ligated and severed, body weight was obtained, and the lamb was transferred to a heated surgical bed under a radiant warming lamp. The arterial catheter was connected to a transducer for pressure monitoring and the venous catheter was connected to a pump for continuous infusion of 5% dextrose in water (D5W) at a rate of 6 ml/kg/hr. A rectal thermometer was inserted to monitor body temperature and the lamb was covered with plastic to minimize evaporative heat loss. The airway was suctioned and 2.5 ml/kg surfactant (Curosurf®, Chiesi Farmaceutici S.p.A., Parma, Italy) was administered intratracheally over a period of five minutes in four positions (Trendelenburg, reverse Trendelenburg, and left and right lateral decubitus positions). Lambs were to receive four mechanical breaths for lung recruitment and then started on CPAP via the endotracheal tube initiated at 5 cmH2O and continuously optimized to effect (4-8 cmH2O) along with a fraction of inspired oxygen (FIO2) over the course of the study. 

After a stabilization period of 15 minutes from birth, the protocol called for the first lamb to be started on a continuous infusion of ENA-001, with ascending dose hourly, while the second lamb was to serve as a sham (D5W) control. The ENA-001 treatment was designed to be started without a loading dose such that plasma concentrations would climb over the course of each hour and contribute to an ascending dose effect. The four hourly doses are described in Table 1. The first three doses have been used in human studies of efficacy, where the lower dose had minimal effect, the second dose is the intended adult therapeutic dose, the third dose is the intended adult human loading dose, and the last hourly dose rate is given to support toxicology analysis in the immature tissues as it is 10-fold the intended adult therapeutic dose. 

**Table 1:** Doses of ENA-001 and rationale for the four one-hour periods after delivery

Hour	Dose	Description	Purpose
1	0.4 mg/kg/hr	Low dose studied in adult humans; minimal efficacy	To assess if the premature lambs were more responsive than adult humans
2	1.1 mg/kg/hr	Dose being studied in adult humans as efficacious	To evaluate comparability
3	2.0 mg/kg/hr	Loading dose used in adult humans	To evaluate comparability
4	12 mg/kg/hr	10x therapeutic dose	To support a toxicology assessment

Pulmonary function assessments were to be conducted every 15 minutes, along with a recording of vital signs and arterial blood draws for arterial blood gas and samples for ENA-001 concentrations. For pulmonary function assessments, a pneumotach was placed between the endotracheal tube and the CPAP connector. At least 10 representative breaths free of artifacts from motion or atypical breaths were recorded using a pulmonary function system designed for neonatal research (NewLifeBox-R, Advanced Life Diagnostics, Weener, Germany). For each period, the rate, tidal volumes, and minute ventilation were derived from an average of the 10 breaths. 

To maintain a stable plane of anesthesia, repeat doses of fentanyl (1 µg IM) were given as needed throughout the protocol based on blood pressure response to stimulation. At the conclusion of the study, animals were sacrificed and tissue samples were harvested for subsequent histopathology. 

## Results

Two male lambs were delivered, weighing 3.2 kg (test animal) and 4.1 kg (sham animal). Instrumentation was uneventful; however, neither lamb exhibited a drive for spontaneous breathing. Each animal required manual ventilation throughout the entire stabilization period and beyond, with a complete absence of spontaneous effort. The inability to stimulate breathing with resuscitation efforts was unexpected given the late gestational age selected. Therefore, based on the treatment assignment, each animal had a unique clinical course. 

Clinical viability: test animal 

Despite the poor prognosis owing to the absence of ventilatory effort, continuous infusion of the first dose of ENA-001 was started 20 minutes following birth. The test animal continued to require manual ventilation, which was continued for an additional 10 minutes. 

As the stated purpose of this study was to validate the model, the decision was made to provide a loading dose as an intravenous (IV) bolus infusion of ENA-001. One mL of the stock solution of ENA-001 (10 mg/ml) was drawn up and infused through the venous catheter. Given that the lamb weight of 3.2 kg, this bolus was 3.1 mg/kg, which was selected because 3 mg/kg is a bolus IV injection that has yielded transient therapeutic plasma levels in animal studies previously conducted to develop the bolus use of ENA-001 (unpublished preclinical development work being done by Enalare: Dose Toxicity/Toxicokinetic Study in Rats with a 14-Day Dose-free Recovery. February 11, 2022).

Nearly instantaneously following the delivery of the IV bolus, the lamb began breathing spontaneously and never again required manual intervention for the remainder of the study. The CPAP system was connected to the endotracheal tube and pressure was decreased to 4 cmH2O. For the remainder of the four hours following the initiation of the first infusion dose, the doses were changed hourly as per the protocol as shown in Table 1. The radiant heat was adjusted to maintain temperature and physiologic data were recorded every 15 minutes per the protocol. At four hours following the initiation of continuous infusion, the animal was sacrificed for tissue harvest. 

Clinical viability: sham animal 

The sham animal was delivered approximately an hour following the test animal. This delay was attributed to the stabilization period of the test animal requiring additional time and personnel involvement. The sham animal was delivered and instrumented in the same manner as described for the test animal. As with the test animal, the sham animal lacked spontaneous breathing efforts. A decision was made to manually ventilate for 30 minutes to match the course for the test animal, and if still not breathing, would be determined inviable and sacrificed for the control tissue samples. 

At the 30-minute time point, again considering the purpose of this study, a 1 mL IV bolus infusion of ENA-001 (10 mg/mL) was delivered. At this lamb’s body weight, the bolus was a lesser 2.4 mg/kg. Nearly instantaneously following the delivery of the IV bolus, the lamb began breathing spontaneously. After several minutes, the spontaneous breathing efforts abated, and manual ventilation was resumed. The animal was then sacrificed for tissue harvest. 

Physiology in the treatment animal 

**Figure 1:**
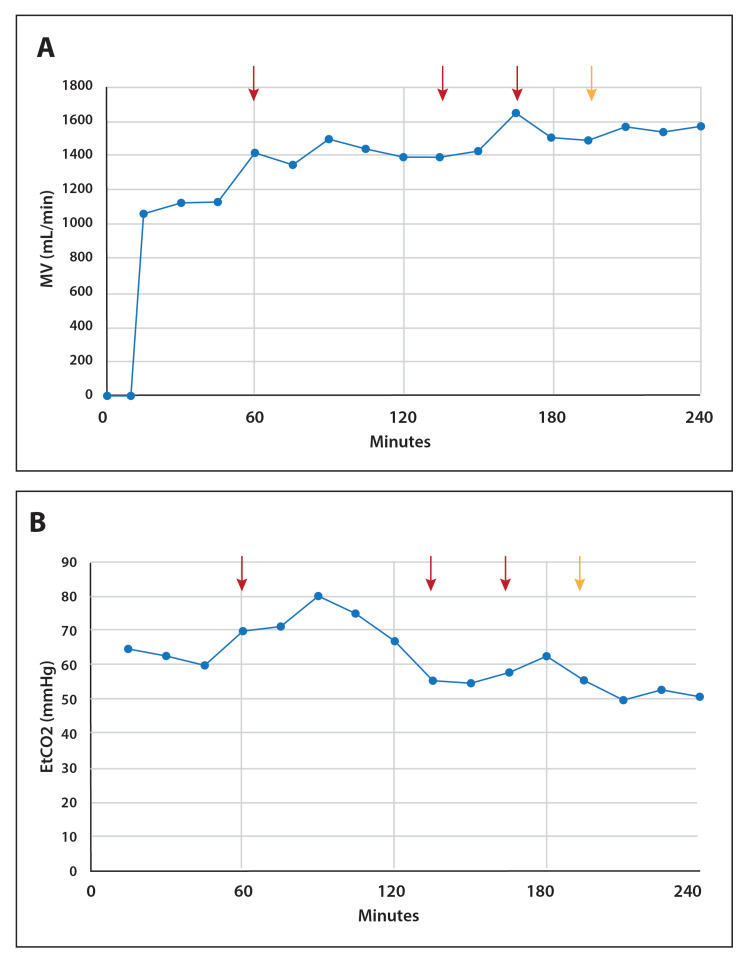
MV and EtCO2 in response to ascending dose of continuous infusion ENA-001 MV continued to rise throughout the four-hour ascending dose protocol following the bolus injection of ENA-001, associated with a concomitant drop in EtCO2.  The arrows (red and yellow) depict the point where 1 µg of fentanyl was injected intravenously for anesthesia.  The yellow arrow designates where the breathing effort became clearly more abdominal. MV: minute ventilation; EtCO2: end tidal carbon dioxide level

**Figure 2:**
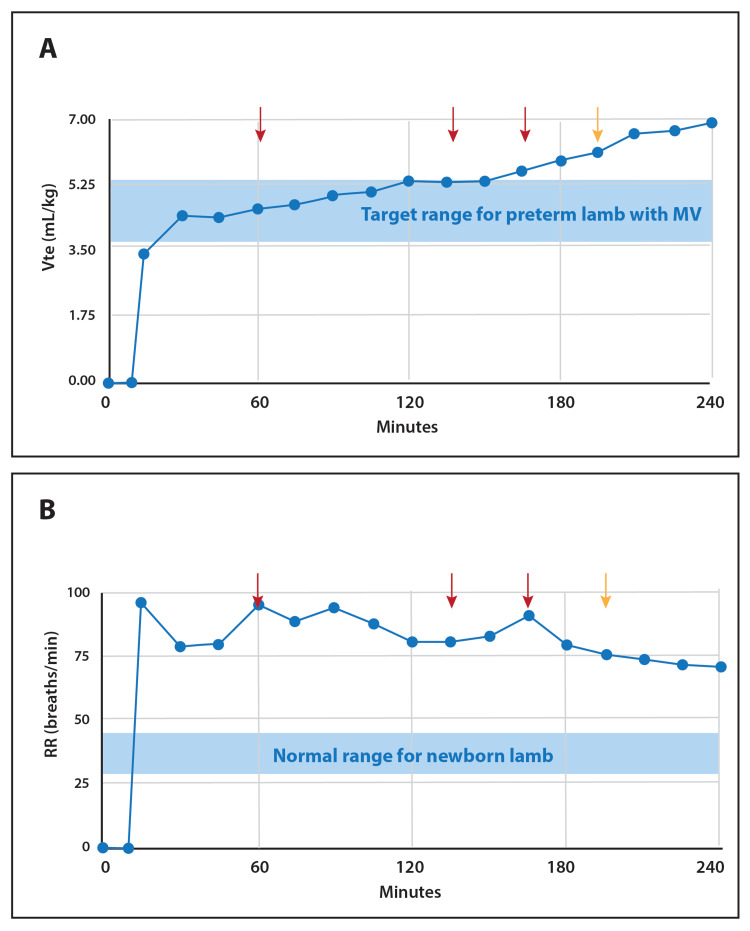
Breathing mechanics in response to ascending dose of continuous infusion ENA-001 (A) Vt rose consistently throughout the four-hour ascending dose protocol. The spontaneous efforts exceeded the target range in mL/kg for mechanical ventilation of preterm lambs in the literature [14]; (B) RR appeared stable with a downward trend, and appeared to be most reactive to fentanyl administration.  The arrows (red and yellow) depict the points where 1 µg of fentanyl was injected intravenously for anesthesia.  The yellow arrow designates where the breathing effort became clearly more abdominal.  RR was consistently well above the range for a newborn lamb [13] and above the upper limit reported in the literature for management with mechanical ventilation [14]. Vt: tidal volume; RR: respiratory rate

**Figure 3:**
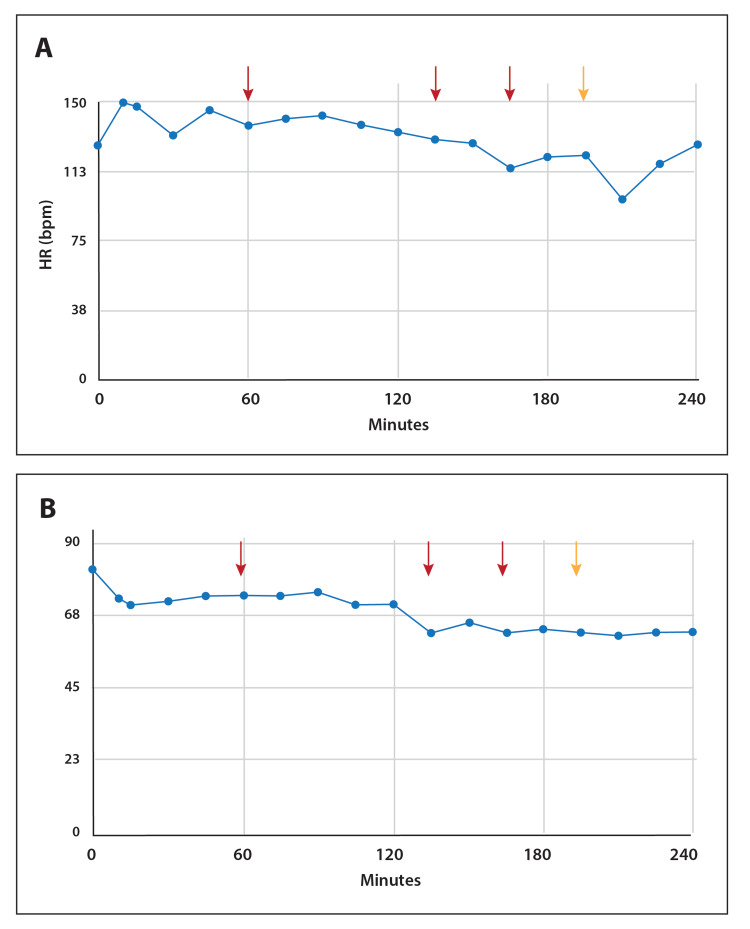
Hemodynamics in response to ascending dose of continuous infusion ENA-001 HR (A) and MAP (B) were stable throughout the four-hour period. The arrows (red and yellow) depict the points where 1 µg of fentanyl was injected intravenously for anesthesia.  Either HR and/or MAP appeared to respond to each fentanyl injection with a downward deflection. The yellow arrow also designates where the breathing effort became clearly more exaggerated. HR: heart rate; MAP: mean arterial pressure

The treatment lamb required six 1 µg (0.31 ug/kg) bolus intramuscular injections of fentanyl to maintain stable anesthesia per the protocol. The first dose of fentanyl was given at delivery, four doses of fentanyl were given during the four-hour experimental period and a sixth dose was given just before sacrifice. The timing of the four fentanyl doses given during the experimental period is identified by arrows in Figures 1, 2, 3 and is aligned with inflection points in respiratory rate (RR), tidal volume (Vt), or breathing pattern. At baseline (end of stabilization period and start of infusion) and for 10 minutes after, spontaneous minute ventilation (MV) was zero (Figure 1A). Immediately following the bolus loading dose, spontaneous MV immediately changed from zero to approximately 1,100 mL/min. MV continued to rise through the course of the four hours with a concomitant decrease in end tidal carbon dioxide level (EtCO2) (Figure 1B). 

ENA-001 had the most dramatic effect on Vt, where a consistent rise was seen through the four hours (Figure 2A). Reported as Vt per kg of body weight (Figure 2A), spontaneous breaths were at least as deep and ultimately surpassed the lung protective range of 4-6 mL/kg. RR gradually decreased over the four hours and was more acutely reactive to the fentanyl delivery (Figure 2B). Nonetheless, RR was always substantially greater than the normal breathing rate for newborn lambs and indicated a large contribution to MV [13].

Figure 3 shows the trends in heart rate (HR) and mean arterial pressure (MAP) over the four hours. Both indices of hemodynamics were stable. Such that HR and/or MAP demonstrated a downward deflection around the time of each fentanyl injection, these data appear to reflect the acute and cumulative effects of fentanyl. 

Blood arterial oxygen saturation (SpO2) was recorded between 97% to 100% except for three measurement periods where the pulse oximetry signal was unattainable or unreliable. Due to a technical issue, the blood gas analyzer was not functioning on the day of the experiment and, therefore, the blood chemistry data were not captured.

## Discussion

The objective of this proof-of-concept study was to verify the prospect that ENA-001 would drive ventilation in the immature ventilatory circuitry before larger studies are designed. The expectation was that an ascending dose scheme in the treatment animal would yield a respectively increasing dose response, substantially differentiating the treatment lamb from the matched twin control. 

Following bolus administration of a loading dose of ENA-001, the effects were clear and profound, taking the animals from absolutely no spontaneous breathing effort to a complete, uninterrupted, and effective spontaneous effort in both lambs. The vigorous respiratory effort continued without abatement in the lamb receiving continuous infusion of ENA-001, even at the lowest dose, a dose which has shown limited efficacy in adult humans to augment an existing ventilatory drive. The bolus dose in the sham control animal demonstrated the transient effect of the bolus delivery and is consistent with the known rapid onset and clearance of ENA-001 from unpublished non-clinical pharmacodynamics studies (Lisa Bernard, MS (Frontage Laboratories Inc). ENA-001: Acute Bolus Intravenous Single).

Despite the inability to measure arterial blood gases due to a technical issue with the instrument, EtCO2 demonstrated a progressive decline. It should be taken into account that we presume hypercarbia was ensuing during the initial period of manual ventilation prior to delivery of the ENA-001 loading dose, due to poor respiratory drive after birth, immediately during surfactant delivery, with pauses to allow for spontaneous efforts to surface and an inability to monitor effective ventilation in real-time. This was reflected by EtCO2, which was elevated from the start. Moreover, compared to existing data on ventilation in the neonatal lamb, the current ventilation data suggest at least effective, sufficient spontaneous ventilation during ENA-001 delivery. The normal range for RR in the newborn lamb is between 36 to 48 breaths per minute [13] and prior literature on the management of preterm lambs by mechanical ventilation limits breaths per minute to not exceed 70 [14]. In the current study, RR was never below 70 breaths per minute. Moreover, in the prior literature on mechanical ventilation of the lamb, Vts were limited to between 4-6 mL/kg [14], which combined with the maximal allowable RR (70 breaths/min) would yield a maximal MV value for this lamb to be 1,344 mL/min. However, the lamb in this study had an MV that exceeded 1,344 mL/min for all but the first three measurements of spontaneous breathing and was always within or above the noted target tidal volume range. Hence, the lamb was most likely hyperventilating by the end of the study period, driven by the higher doses of ENA-001. 

Additionally, the test animal required an appreciable quantity of fentanyl throughout the course of the study. Notwithstanding, the heightened breathing drive continued, despite the acute effects on breathing rate and the cumulative exposure to fentanyl. Into the fourth hour of the experimental period, when the animal was receiving the supratherapeutic dose, a notable shift to abdominal breathing was observed, for which the fentanyl dose was given at 3.25 hours mark. At this point, tidal volumes exceeded the target range noted above. This period is confounded by several factors that need to be elucidated in further studies, including hyperventilation induced by a very high dose of ENA-001, fatigue from prolonged robust breathing efforts, and the cumulative effects of 1.6 µg/kg of fentanyl in under four hours. 

When interpreting the data, it should be noted that there were no loading doses intended for each of the four infusion doses defined in the protocol. It was expected that 134-day gestation lambs would have a baseline breathing effort. As such, it is not surprising that the initial ENA-001 infusion did not stimulate breathing without the addition of an initial bolus, as this first, low dose would take appreciably longer than 10 minutes to achieve an effective plasma concentration. Therefore, the injection of a bolus loading dose was a real-time modification necessary to salvage the model. Additionally, given the lack of loading doses coupled with the subsequent ascending doses over the last three hours, it was not expected to see clear plateaus in effect, but rather a more continuous rise in response. Therefore, these data on respiratory parameters sufficiently demonstrate as a proof-of-concept that ENA-001 will stimulate the immature neural respiratory circuitry in the preterm lamb model. 

The primary limitation of this study is the small sample size. That said, the learnings from this study have already proven useful to revise and support protocols for the ongoing research to better elucidate the effect of ENA-001 on neonatal physiology. Another considerable limitation is the lack of arterial blood gas data due to a technical malfunction of the system; hence, we rely on ETCO2 and SPO2 to gauge the effectiveness of ventilation and oxygenation. A further limitation was the effect that fentanyl demonstrated on the breathing pattern. While having a noticeable impact on the primary outcomes parameters, as expected for a strong opioid, it was noteworthy to see the limited degree of this impact and the apparent refractory dose effect to ENA-001. The ability to reverse opioid-induced respiratory depression has been demonstrated in human and non-clinical models with ENA-001 [9,15]. Regardless, these results have led to an IACUC-approved revision of the sedation and analgesia regimen used for the lambs in subsequent studies.

## Conclusions

In conclusion, this proof-of-concept study demonstrates that ENA-001 has a profound effect on the ventilatory drive in the neonatal lamb. These results suggest that ENA-001 might be an effective therapy, alone or as comedication, for the treatment of AOP. They further suggest that ENA-001 might have broader applications in situations of neurological ventilatory insufficiency. Further study is now warranted to elucidate optimal dose range as well as interactions with GA and caffeine administration. 
